# Epidemiological and molecular characterization of *Streptococcus pneumoniae* carriage strains in pre-school children in Arkhangelsk, northern European Russia, prior to the introduction of conjugate pneumococcal vaccines

**DOI:** 10.1186/s12879-020-04998-5

**Published:** 2020-04-15

**Authors:** V. Vorobieva S. Jensen, A-S Furberg, H-C Slotved, T. Bazhukova, B. Haldorsen, D. A. Caugant, A. Sundsfjord, P. Valentiner-Branth, G. S. Simonsen

**Affiliations:** 1 Research Group for Host-Microbe Interaction, Department of Medical Biology, Faculty of Health Sciences, UiT – The Arctic University of Norway, Tromsø, Norway; 2 Department of Virus and Microbiological Special Diagnostics, Division of Infectious Disease Preparedness, Statens Serum Institut, Artillerivej 5, DK-2300 Copenhagen S, Denmark; 3 Faculty of Health and Social Sciences, Molde University College, Molde, Norway; 4 Department of Microbiology and Infection Control, University Hospital of North Norway, Tromsø, Norway; 5 Department of Bacteria, Parasites and Fungi, Division of Infectious Disease Preparedness, Statens Serum Institute, Copenhagen, Denmark; 6 Department of Clinical Biochemistry, Microbiology and Laboratory Diagnostics, Northern State Medical University, Arkhangelsk, Russia; 7 Norwegian national advisory unit on detection of antimicrobial resistance, Department of Microbiology and Infection Control, University Hospital of North Norway, Tromsø, Norway; 8 Division of Infection Control and Environmental Health, Norwegian Institute of Public Health, Oslo, Norway; 9 Infectious Disease Epidemiology and Prevention, Statens Serum Institut, Copenhagen, Denmark

**Keywords:** PCV-13-vaccine, Pandemic clones, Serotyping, Sequencing, Multidrug-resistance, Russia

## Abstract

**Background:**

The 13-valent Pneumococcal Conjugate Vaccine (PCV-13) was introduced in the National Immunization Programme (NIP) schedule in Russia in March 2014. Previously, the 7-valent Pneumococcal Conjugate Vaccine (PCV-7) was marketed in Russia in 2009 but has never been offered for mass vaccination. A carriage study was performed among children in Arkhangelsk in 2006. The objective was to determine the prevalence of carriage, serotype distribution, antimicrobial susceptibility and the molecular structure of *Streptococcus pneumoniae* strains before marketing and introduction of PCV-13.

**Methods:**

A cross-sectional study was conducted on a cluster-randomized sample of children and a self-administrated questionnaire for parents/guardians.  Nasopharyngeal samples were collected from 438 children younger than 7 years attending nurseries and kindergartens in the Arkhangelsk region, Russia. Detailed demographic data, as well as information about the child’s health, traveling, exposure to antimicrobials within the last 3 months and anthropometric measurements were collected for all study subjects. Variables extracted from the questionnaire were analysed using statistic regression models to estimate the risk of carriage. All pneumococcal  isolates were examined with susceptibility testing, serotyping and multilocus sequence typing.

**Results:**

The overall prevalence of asymptomatic carriage was high and peaking at 36 months with a rate of 57%. PCV-13 covered 67.3% of the detected strains. High rates of non-susceptibility to penicillin, macrolides and multidrug resistance were associated with specific vaccine serotypes, pandemic clones, and local sequence types. Nine percent of isolates represented three globally disseminated disease-associated pandemic clones; penicillin- and macrolide-resistant clones Norway^NT^-42 and Poland^6B^-20, as well as penicillin- and macrolide-susceptible clone Netherlands^3^-31. A high level of antimicrobial consumption was noted by the study. According to the parent’s reports, 89.5% of the children used at least one antimicrobial regime since birth. None of the hypothesised predictors of *S. pneumoniae *carriage were statistically significant in univariable and multivariable logistic models.

**Conclusions:**

The study identified a high coverage of the PCV-13-vaccine, but serotype replacement and expansion of globally disseminated disease-associated clones with non-vaccine serotypes may be expected. Further surveillance of antimicrobial resistance and serotype distribution is therefore required.

## Background

*Streptococcus pneumoniae* is a bacterial pathogen causing disease among all age groups. Despite the introduction of effective vaccines, invasive pneumococcal disease (IPD) is associated with high mortality and morbidity [1–3]. As was anticipated, the introduction of the pneumococcal conjugate vaccines (PCVs) in the national immunization programmes has substantially reduced pneumococcal-related deaths worldwide [4]. Immunization by conjugate pneumococcal vaccines has now been implemented in 145 countries [5]. Still, according to the most recent report based on data from the World Health Organisation (WHO) and the Maternal and Children Epidemiology Estimation collaboration*,* in 2015, pneumococci were estimated to have caused 318,000 (uncertainty range 207,000–395,000) deaths for both HIV-infected and HIV uninfected infants and young children in the age 1–59 months globally [4].

The epidemiology of pneumococcal disease prior to the introduction of pneumococcal vaccines was dominated by the spread of global disease-causing epidemic clones, both multidrug-resistant (MDR) and antimicrobial susceptible clones [6]. The success of epidemic clones, though not well understood, has been linked to certain capsular types [7, 8], carriage of a pilus islet [9] and various virulence factors [10]. Mass vaccination has reduced the occurrence of MDR Pneumococcal Molecular Epidemiology Network (PMEN) clones with serotypes covered by the vaccine. Reports from countries dating to the post-PCV era show a rapid reduction of PCV-serotype-related PMEN-isolates. However, some sequence types (ST) ST320, ST433, ST191 and other highly successful clones with non-vaccine related serotypes rapidly replace the disease-associated endemic clones shortly after the introduction of PCV-vaccines [11]. Capsule serotype replacement in clones targeted by PCVs has also been demonstrated [12], such as a switch from 19F to 19A in the disease-associated high-level penicillin- resistant endemic clone Taiwan^19F^-14 [13].

Russia is a large country with an estimated infant and child population aged up to 4 years of 9.0 million in 2019 [14]. The immunization programme for infants and children in the Russian Federation presently includes ten less expensive vaccines, while, for example, the *Haemophilus influenzae* type b conjugate vaccine has not been available for mass vaccination [15]. The PCV-7 was marketed in Russia in 2009 but has never been offered for mass vaccination. The extended pneumococcal conjugate vaccine with 13 serotypes was licenced in Russia in 2011 (PCV-13, Prevenar 13, Wyeth Pharmaceuticals Inc., marketed by Pfizer Inc.), and included in the Russian National Immunization Programme (NIP) schedule in March 2014 [5, 15]. Immunizations are administered in a 2 + 1-dose schedule, with two primary immunizations given at 2 and 4.5 months and a booster at 15 months of age [15]. No additional catch-up immunization has been offered for the rest of the child population [15]. National immunization coverage data are only partially available, but a sharp increase of PCV-coverage was reported by the WHO/ United Nations International Children’s Emergency Fund reporting system in the 3 years after the introduction [16]. In 2017, the rates of PCV-13 coverage were 88 and 70% for the 2nd and the 3rd doses, respectively, while the rates for the 1st dose remain unknown since 2014 [16].

Neither national nor regional surveillance of incidence for IPD cases exists in Russia [17, 18] . The overall incidence of pneumococcal meningitis in Russia was estimated at 0.2 per 100,000 cases for all age groups, and 18% of all cases were presented by children under 5 years. The low rates of pneumococcal meningitis have been associated with suboptimal diagnostics and antimicrobial treatment preceding laboratory examinations [17, 18].

The present study was conducted in the Arkhangelsk region in the northwest part of Russia where no data about the pre-PCV carriage are available. In order to determine pneumococcal carriage at baseline [19] and evaluate possible effects of the introduction of PCV-13 in the Russian immunization schedule, the authors performed a cross-sectional study of asymptomatic nasopharyngeal *S. pneumoniae* carriage in healthy pre-school children attending daycare centres (DCCs) 8 years before the introduction of PCV-13. All pneumococcal isolates were analysed with regard to serotypes, phenotypic antimicrobial resistance patterns and population structure based on multilocus sequence typing (MLST).

## Methods

### Study population

Children and parents/guardians from ten DCCs were invited to participate in the study. All the DCCs were public childcare institutions that belonged to small towns and suburbs of the Arkhangelsk region and located within a range of 13 to 44 km from the city of Arkhangelsk. Each DCC consisted of a nursery and a kindergarten and was attended by 21 to 200 children. Besides, 32 randomly chosen healthy children living in the centrum of Arkhangelsk were sampled. Children and parents/guardians were invited to participate by the announcement in the local newspaper. All children were sampled during the last week of November 2006 by one otolaryngologist. None of the children experienced symptoms of a common cold like cough, runny or stuffy nose at the date of examination. The body temperature was normal for all children on the day of sampling.

The questionnaires were filled out by parents or guardians for all participants in the study. Each questionnaire included questions concerning the child’s health, length of breastfeeding, travelling abroad or outside of the Arkhangelsk region within the last 6 months, smoking habits of family members, as well as the household size and the number of siblings and family members. Information on the use of antimicrobials agents during the last 3 months prior to sampling was also collected for all children. Anthropometric measurements were taken for all participants on the day of sampling.

All parents/guardians were informed about the study by informational letters and a majority of the parents/guardians participated in informational meetings. The written informed consents were filled out by parents or guardians for all participants of the study. Ethical approval was obtained from the Ethics Research Committee of North Norway, the reference number of the approval 5.2006.2086 from the 23rd of June 2006 and the Ethics Research Committee of the Northern State Medical University of Arkhangelsk, the reference number for the approval 06/06 from the 6th of June 2006. Permission to conduct the study was also obtained from the Health and Educational Services of the Arkhangelsk Region.

### Bacterial identification and serotyping

The European Intervention Study (EURIS) manual was used for isolation of bacterial strains [20, 21]. Nasopharyngeal samples were transferred to the laboratory using transport media swabs (Copan 114C, Copan Diagnostics, Inc., Corona, USA), and inoculated within 3 to 6 h after the arrival. Samples were cultured on 5% defibrinated sheep blood agar (Oxoid Ltd., UK) supplemented with gentamicin (5 mg/L) and incubated at 35–37 °C under anaerobic conditions for 18 to 24 h. Samples were also cultured on sheep blood agar (Oxoid Ltd., UK) with optochin disks (AB Biodisk, Sola, Sweden) (5 μg) and incubated in 5% CO_2_ at 35–37 °C for 18–24 h. Strains were identified as *S. pneumoniae* by colony morphology, negative catalase reaction, optochin susceptibility, agglutination in the Pneumo-Kit slidex test (BioMèrieux, Missouri, USA), and by the bile solubility test [22]. Isolates were serotyped by the Quellung reaction using serotype-specific antisera (SSI Diagnostica, Denmark).

### Antimicrobial susceptibility testing

Strains were tested for antimicrobial susceptibility by disk diffusion on Iso-Sensitest Agar (ISA) (Oxoid Ltd., Basingstoke, UK) supplemented with nicotinamide adenine dinucleotide (Mast Diagnostics Merseyside, UK) and 5% defibrinated sheep blood. Antimicrobial paper disks (Oxoid Ltd., UK) containing 1 μg oxacillin (OXA), 15 μg erythromycin (ERY), 30 μg tetracycline (TET), 25 μg trimethoprim-sulfamethoxazole (SXT) or 10 μg norfloxacin (NOR) were used. OXA-resistant isolates (inhibition zone < 18 mm) were further examined by penicillin G (PEN), cefuroxime (CXM), cefotaxime (CTX) and meropenem (MEM) Etests according to the manufacturer’s instructions (AB Biodisk). Unless otherwise stated, the breakpoints defined by the Norwegian Working Group for Antibiotics (NWGA) were used. NOR-resistant isolates were examined by Etest for their susceptibility to ciprofloxacin (CIP), NOR, moxifloxacin (MXF) and levofloxacin (LVX), using breakpoints from the Swedish Reference Group for Antibiotics (SRGA) [23–25]. Multidrug-resistance (MDR) was defined as resistance to three or more antimicrobial  classes [26].

The double-disk diffusion (DDD) test with ERY and clindamycin (CLI) (Oxoid Ltd., UK) was used for characterization of inducible macrolides, lincosamides, streptogramines (iMLS) resistance as described [27]. Blunting of the CLI inhibition zone indicated iMLS_B_-resistance, resistance to both ERY and CLI indicated constitutive resistance (cMLS_B_), whereas susceptibility to CLI and resistance to ERY indicated M-type resistance. Detection of the macrolide resistance determinants *ermB* and *mefA* was performed by PCRs as previously described [28].

### Multilocus sequence typing and comparative analysis

All strains were examined by MLST as described by Enright et al. [29] and assigned to STs based on a combination of alleles at seven housekeeping loci. The seven housekeeping genes used for MLST were *aroE, gdh, gki, recP, spi, xpt,* and *ddl*. Alleles were identified and isolates were assigned into STs using the PubMLST database (https://pubmlst.org/spneumoniae/). The PHYLOViZ® programme was used for assigning the isolates into clonal complexes (CCs), defined as clusters sharing six out of seven common alleles.

### Data analysis

RStudio© version 1.1.423 {https://www.rstudio.com/} and R version 3.5.1 for Windows were used for calculation of odds ratios (OR), confidence intervals (95% CI), and *p*-values using two-tailed Fisher’s exact test. Carriage rates were calculated as incidence rate ratios (IRRs) with 95% CI. *P*-values < 0.05 were considered significant.

We used Fisher’s exact test and univariable and multivariable logistic regression models to examine potential risk factors for pneumococcal carriage including sex, age, early life variables (weight and length at birth, breastfeeding length, living in Arkhangelsk since birth), family and socioeconomic status (parents’ education, having siblings < 5 years at the moment of examination, number of rooms at home), medication and disease (having had rhinitis, otitis or pneumonia since birth, average number of respiratory tract infections per year since birth, regular medication, any disease within a month prior to the examination, receiving antimicrobials within 3 months prior to the examination), and lifestyle factors (body height, body weight, Body Mass Index (BMI), passive smoking).

## Results

### Characteristics of the study population and overall carriage rate

Out of 766 children attending the ten selected DCCs, 438 (57.2%) agreed to participate in the study and nasopharyngeal swabs were collected from all these children. The demographic data for all 438 children are given in Table 1. The percentage of parents or guardians who agreed to the participation of their children varied from 33.6 to 88.5% between institutions and the number of isolates ranged from 2 to 51 between institutions. Samples were gathered from non-vaccinated healthy children aged 6 to 83 months (mean age 49.3 months), and 51.1% of the children were boys. BMI was in the range between 13 and 16 kg/m^2^ for 56% of the children and greater than 16 for 43% of the children on the day of examination. Only 1% of the children had a BMI of less than 13 on the day of sampling. The overall prevalence of antimicrobial consumption was high. According to the parent’s reports, 89.5% of the children used at least one antimicrobial regime since birth. Additionally, 38.7% (65/168) of the children with recognized carriage have been treated with antimicrobials within the last 3 months before sampling.

**Table 1:** Potential risk factors in the study population of *Streptococcus pneumoniae* nasopharyngeal carriage status. A study among children in daycare centers in Arkhangelsk, Russia 2006, *N* = 438**ª**. The table displays the number of carriers, total number of examined children, carriage rates, confidence intervals (CI) and *p*-values

Risk factors	Variable	Number of carriers (total number of children)	Carriage rate % (95% CI)	P-value^b^
Sex	Female	90 (214)	42.1 (35.4–48.7)	1.00
	Male	78 (224)	34.8 (28.6–41.1)	0.14
Age, months	≤18	1 (12)	8.3 (7.30–24.0)	1.00
	19 - ≤36	55 (109)	50.5 (41.1–59.8)	0.02
	37 - ≤59	73 (188)	38.8 (31.6–45.8)	0.06
	≥60	39 (129)	30.2 (22.3–38.2)	0.01
Birth weight, g	< 2500	11 (30)	36.7 (19.4–54.0)	1.00
	≥2500	146 (384)	38.0 (33.1–42.9)	0.88
Birth height, cm	< 48	10 (20)	50.0 (28.1–71.9)	1.00
	≥48	146 (397)	36.8 (32.0–41.5)	0.24
Breastfeeding	None or < 3 months	32 (62)	51.6 (39.2–64.1)	1.00
	≥3 months	136 (208)	65.4 (58.9–71.8)	0.14
Living in Arkhangelsk since birth	Yes	152 (395)	38.5 (33.7–43.3)	1.00
	No	11 (37)	29.7 (15.0–44.5)	0.38
Any disease within a month prior study	Yes	115 (302)	38.1 (32.6–43.4)	1.00
	No	52 (131)	39.7 (31.3–48.1)	0.75
Average number of respiratory tract infections per year since birth	Never or rare	31 (88)	33.7 (23.9–43.5)	1.00
	Less than 6 times per year	77(211)	37.5 (30.9–44.0)	0.76
	More than 6 times per year	58 (135)	42.2 (33.8–50.6)	0.58
Regular medication	Yes	15 (36)	41.7 (25.6–57.8)	1.00
	No	149 (394)	37.8 (33.0–42.6)	0.72
Highest education parent(s)	*Secondary professional school or higher*	225 (604)	37.3 (33.4–41.1)	1.00
	*Secondary, primary or non-specified*	86 (207)	41.5 (34.8–48.3)	0.31
Sibling(s) < 5 years	Yes	28 (68)	41.2 (29.5–52.9)	1.00
	No	137 (234)	58.5 (50.5–66.6)	0.06
Number of rooms	1–2	92 (262)	35.1 (29.3–40.9)	1.00
	≥3	74 (173)	42.8 (35.4–50.2)	0.47
Passive smoking	Yes	111 (189)	58.7 (51.7–65.7)	1.00
	No	48 (125)	38.4 (29.9–46.9)	0.12

Values are number of subjects (%) if not otherwise stated

ªNumber may vary due to missing data

^b^Confidence intervals (CI) and p-values were calculated by two-tailed Fisher’s Exact Test

A *p*-value < 0.05 was considered significant

### Risk factors

Pneumococci were isolated from 168 children (mean age 45.8 months), giving an overall carriage rate of 38.4% (CI 33.8–42.9%). The highest rate of carriage was found among the children aged from 19 to 36 months and it was lowest at the age less than 18 months (Table 1). The carriage rate peaked at the age of 36 months (57.0%).

The carriage rate of *S. pneumoniae* for males and females were compared with univariable and multivariable regression models to estimate the risk of carriage (odds ratios) and did not display any statistically significant differences. Sex did not influence the carriage rate of *S. pneumoniae* significantly (Table 1). None of the hypothesised predictors of *S. pneumoniae* carriage, including sex, breastfeeding, number of rooms at home, respiratory tract infections and illness, were statistically significant in univariable and multivariable logistic regression models (Table S1). Receiving antimicrobial therapy 3 months before to sampling was not significantly associated with carriage of penicillin non-susceptible *S. pneumoniae* (OR 0.71 with 95% CI 0.29–1.76).

### Serotypes and vaccine coverage

Twenty-four different serotypes were detected in the pneumococcal collection, and 14 isolates were non-typeable (NT) (Table 2). Serotypes 19F (*n* = 28; 16.7%), 23F (*n* = 21; 12.5%), 6A (*n* = 18; 10.7%) and 6B (*n* = 16; 9.5%) were most prevalent (Tables 2 and 3). The most diverse serotype composition was observed in children in the age groups 24 to 35 months and 36 to 47 months with a  total number of 16 different serotypes in each group. The diversity of serotypes in other age groups varied from eight (age group 16 to 23 months) to 13 (age group 48 to 59 months). Most isolates (5/6, 83.3%) with serotype 14 were found in samples taken from children aged less than 48 months of age.

**Table 2:** Serotype distribution of *Streptococcus pneumoniae* carriage isolates (*N* = 168) among 438 children in daycare centers in Arkhangelsk, Russia, 2006. Proportion of isolates covered by three different pneumococcal vaccines PCV-10, −13 and PPV-23. Penicillin non-susceptible and macrolide non-susceptible strains, as well as strains with combined non-susceptibility to penicillin and macrolides are specified

Serotypes	No.	PCV-10 1, 4, 5, 6B, 7F, 9 V, 14, 18C, 19F, 23F	PCV-13 1, 3, 4, 5, 6A/B, 7F, 9 V, 14, 18C, 19F, 23F	PPV-23 1, 2, 3, 4, 5, 6B, 7F, 9 N/V, 10A, 11A, 12F, 14, 15B, 17F, 18C, 19A/F, 20, 22F, 23F, 33F	PNSP^**a**^	MNSP^**b**^	PMNSP^**c**^
6B	16	16	16	16		4	3
9 V	4	4	4	4			
14	6	6	6	6		1	
18C	9	9	9	9			
19F	28	28	28	28			
23F	21	21	21	21	7		
3	11		11	11			
6A	18		18				
11A	8			8			
9 N	4			4			
15B	4			4			
22F	1			1			
7C	1						
13	3						
15A	2						
15C	4						
16F	1						
18F	1						
23A	4					1	
31	1						
35B	1						
35C	1						
35F	2					1	
38	3						
NT^d^	14				3	1	5
Total number of strains	168	84	113	112	10	8	8

^a^Penicillin non-susceptible pneumococcal strains

^b^Macrolide non-susceptible pneumococcal strains

^c^Penicillin and macrolide non-susceptible pneumococcal strains

^d^Non-serotypeable

**Table 3:** Distribution of 24 clonal complexes and non-related isolates of *S. pneumoniae* circulating in the Arkhangelsk region in 2006 in conjunction to MLST sequence types, non-susceptibility to penicillin/macrolides, serotypes and relationship to global pandemic Pneumococcal Epidemiology Network (PMEN) clones. Sequence types with corresponded serotypes identical with three of the 43 PMEN clones are highlighted in a bold font

CC^a^ No(%)	ST^b^	No	PNSP/MNSP	Serotype	Relationship to PMEN-strains
CC15 15(8.9)	25	1	M^c^	14	SLV^g^ CSR^14^–10, DLV England^14^–9.
	423	9		19F	DLV^h^ CSR^14^–10, DLV England^14^–9
	2995	3		14	DLV CSR^14^–10, DLV England^14^–9
	2997	2		14	TLV^i^ CSR^14^–10, TLV England^14^–9, TLV CSR^19A^-11
CC62 4(2.4)	62	4		11A/NT	SLV Netherlands^8^–33
CC66 3(1.8)	3104	1		9 N	DLV Tennessee^14^–18
	3224	2		9 N	SLV Tennessee^14^–18
CC102 9(5.4)	102	7		18C	
	1016	1		18C	
	3187	1		18C	
CC113 2(1.2)	1363	1		35C	DLV Netherlands^18C^-36
	3107	1		6B	SLV Netherlands^18C^-36
CC123 3(1.8)	123	3		13	SLV Netherlands^18C^-36
CC124 13(8.3)	2994	13		23F	
CC180 11(6.5)	**180**	2		**3**	**Netherlands**^**3**^**–31**
	505	8		3	DLV Netherlands^3^–31
	3202	1		3	TLV Netherlands^3^–31
CC193 2 (1.2)	2186	2		15A	TLV^i^ Greece^21^–30
CC271 6(3.6)	2993	3		19F	SLV of Taiwan^19F^-14
	2998	3		19F	SLV of Taiwan^19F^-14
CC315 7(3.6)	**315**	2	P and M^d^	**6B**	**Poland**^**6B**^**-20**
	**315**	3	M	**6B**	**Poland**^**6B**^**-20**
	3200	1	M	6B	SLV Poland^6B^-20
CC344 9(5.4)	**344**	7	P and M	**NT**^f^	**Norway**^**NT**^**-42**
	**344**	1	M	**NT**	**Norway**^**NT**^**-42**
	2996	1	P^e^	NT	SLV of Norway^NT^-42
CC393 3(1.8)	393	3		38	
CC490 6(3.6)	490	1		6A	
	1141	3		6A	
	3244	2		6A/B	
CC600 12(7.1)	600	10		6A	
	3203	2		6A	
CC1012 5 (3.0)	1012	1		11A	
	2860	4		11A	
CC1262 7(4.2)	1262	1		15C	
	2859	6		15B/C	
CC1500 7(4.2)	1500	7	P	23F	
CC2989 9(5.4)	2989	9		19F	DLV Colombia^23F^-26
CC2990 2(1.2)	2990	2		6B	
CC2992 4(2.4)	2992	4		19F	
CC3105 3(1.8)	3105	3		23A	TLV Tennessee ^23F^-4
CC3106 3(1.8)	3106	3		6B	
CC3186 3(1.8)	3186	1	P	NT	
	3186	1		NT	
	3208	1	P	NT	
Non-related isolates					
17 (10.1)					
	386	1	P and M	6B	DLV Poland^6B^-20
	1025	1		15C	
	1028	1		35F	
	1470	1		22F	
	1684	1		31	
	2966	1		16F	
	2991	1	M	35F	
	3185	1		35B	
	3188	1		23F	
	3195	1		NT	TLV Netherlands^7F^-39
	3196	1		6B	
	3197	1		9 N	DLV Netherlands^15B^-37
	3198	1		6B	
	3199	1		6A	TLV Greece^21^–30
	3201	1	M	23A	
	3209	1		7C	
	3243	1		18F	

^a^Clonal complex

^b^Sequence type

^c^Macrolide non-susceptible pneumococci

^d^Penicillin and macrolide non-susceptible pneumococci

^e^Penicillin non-susceptible pneumococci

^f^Non-serotypeable

^g^Single locus variant

^h^Double locus variant

^i^Differences in three loci

PCV-13, including serotypes 1, 3, 4, 5, 6A/B, 7F, 9 V, 14, 18C, 19A/F, and 23F, would cover 67.3% (113/168) of the isolates in the study (Table 2). In contrast, PCV-10 provided coverage for only 50.0% (84/168) of the isolates in our collection. The coverage rate for the 23-valent pneumococcal polysaccharide vaccine (PPV) was 66.7% (112/168).

### Antimicrobial susceptibility

The rates of non-susceptibility were as follows: SXT (*n* = 121; 72%), TET (*n* = 52; 31%), OXA (*n* = 29; 17%), and ERY (*n* = 18; 11%). Characterization of penicillin non-susceptible pneumococci (PNSP) and macrolide non-susceptible pneumococci (MNSP) is given below. Only a single strain was resistant to fluoroquinolones (< 1%). MDR was detected in 19 strains (11%). Only 26% (43/168) of nasopharyngeal carriage strains were susceptible to all examined antimicrobials  (OXA, ERY, SXT and TET).

### Genotypes

The isolates displayed 60 different STs (Table 3). Forty-three STs comprising 138 isolates and representing 89.9% of the entire population were assigned into 24 clonal complexes. Clonal complex (CC) 15 represented by 4 different STs (ST25, ST423, ST2995, and ST2997) was found to be the most prevalent complex (8.9%, *n* = 15). Ten percent of all STs were represented by single isolates. Thirty-five of the 60 STs were identified for the first time in Arkhangelsk.

### Association between genotype and serotype

The majority of the pneumococcal isolates in each ST was related to a single serotype. Two STs, ST2859 and ST3244, were associated with two serotypes. To the authors’ knowledge, an association of ST2859 with both serotypes 15B and 15C, as well as ST3244 to both serotypes 6A and 6B has not been reported before. ST62 was represented by three strains with serotype 11A and a single NT strain.

### Non-susceptibility to penicillin and macrolides in association with genotypes

**Fig. 1:**
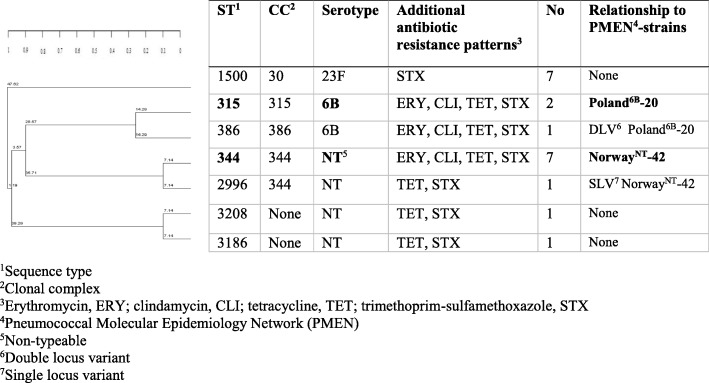
MLST-based phylogeny of 20 penicillin non-susceptible *S. pneumoniae* strains including distribution of serotypes, other resistance patterns than penicillin and relatedness to global pandemic clones isolated in Arkhangelsk, Russia in 2006. Sequence types with corresponded serotypes identical with two of the 43 PMEN clones are highlighted in a bold font

Twenty of 29 (68.9%) OXA-resistant strains were confirmed as non-susceptible to PEN (range 0.094–1 mg/L), and 19 of these strains (95.0%) were MDR. Ten of the 20 (50.0%) isolates were non-susceptible to both PEN and macrolides. The majority of PEN and MNSP isolates (7/10; 70.0%) were related to the Norway^NT^-42 clone (Table 3). Three additional strains expressed serotype 6B and were associated with Poland^6B^-20. The MLST-based phylogeny for all 20 penicillin non-susceptible isolates is given in Fig. 1. The expected rate of PCV-13 coverage for MNSP isolates was 30% (3/10).

Ten pneumococcal isolates displayed non-susceptibility to PEN, but were susceptible to macrolides. Seven of these strains expressed serotype 23F and belonged to ST1500 and were also resistant to STX. The three remaining isolates were non-serotypeable (ST2996; ST3186 and ST3208) (Table 3).

The analysis of all macrolide non-susceptible isolates by DDD method revealed the following phenotypes: iMLS_B_ (*n* = 7), cMLS_B_ (*n* = 4), and M-type resistance (*n* = 5). The results were confirmed by *ermB* and *mefA*-PCRs. Macrolide non-susceptibility was associated with two globally disseminated clones. Six of the macrolide-resistant isolates belonged to CC315 (ST315 and ST3200), expressed the *ermB*-gene and were associated with the international Poland^6B^-20 clone. Six of the isolates belonged to ST344-Norway^NT^-42 and possessed both *mefA* (n = 4) and *ermB* (*n* = 2) determinants. The other 4 macrolide-resistant isolates were represented by unrelated ST25, ST386, ST2991, and ST3201 and were connected to *ermB* (*n* = 3) and *mefA* (*n* = 1) genes.

## Discussion

This is one of the largest carriage surveys prior to the introduction of pneumococcal vaccines in Russia, where serotyping, antimicrobial susceptibility testing and MLST were performed on whole strain collection [30]. Moreover, the present study provides information regarding the population structure of *S. pneumoniae* carriage isolates in pre-school children of the Arkhangelsk region between separately located DCCs. The serotype distribution was diverse in the area, but globally reported epidemiological features, such as age-dependence, dominant serotype prevalence (19F, 23F, 6A/B) and the presence of globally disseminated clones were confirmed in the study.

### Carriage rates

A 38.4% overall frequency of pneumococcal carriage rate was found in non-vaccinated pre-school children. The carriage rate among children aged 36 months was as high as 57.0%. Previous carriage studies in DCCs in Russia have described even higher overall frequencies of asymptomatic *S. pneumoniae* colonization [30–34]. Overall, we found no significant difference in the carriage rates among children with birth weights < 2500 g, birth heights < 48 cm, none or < 3 months of breastfeeding, as well as living with siblings < 5 years. Our study found an average rate of pneumococcal carriage similar to what has previously been described for populations in upper-middle-income countries at a baseline period [35]. The prevalence of carriage is independent of geographical region but strongly associated with accumulated risk factors, such as young age, high-density living conditions, and poor health conditions [36–38].

### Distribution of serotypes

The rates of asymptomatic carriage varied markedly between different age groups in our study, and also the diversity of serotypes displayed age variation. These findings have previously been observed by others [39–41]. Young children aged 19–36 months expressed the highest rates of asymptomatic colonization and the widest range of serotypes. Isolates with serotype 14 (paediatric serotype) were linked to children younger than 47 months in our study. The age-dependence analysis showed a low frequency of pneumococcal colonization up to 19 months and a peak incidence at the age of 36 months with a stable decline from the age of 46 months. This tendency has previously been discovered for children living in developed and upper-middle-income countries [35], but not for children living in low-income countries [42]. The seven most common serotypes (14, 6B, 23F, 19F, 6A, 9 V, 18C) from our study were previously described in the group of the ten most common serotypes of IPD cases globally [43], and they are a part of PCV-13. Three other PCV-13-associated serotypes 1, 5 and 7F are not frequently detected among pneumococcal carriage isolates in Russia [30–32] nor in other geographical areas [40, 41] but were generally related to cases of IPD in infants and young children [44–46].

None of the isolates from our study collection expressed serotype 19A reported as the eighth most prevalent globally [43] and the most common serotype in childhood IPD following PCV-7 introduction [40, 47]. Still, 3.6% of the carriage isolates belonged to the PEN and macrolide-susceptible Taiwan^19F^-14 cluster previously associated with serotype 19F to 19A replacement [13]. Serotype 19A is strongly associated with PEN-resistant cases of IPD and was commonly described shortly after the introduction of PCV-7 in vaccination schedules [48, 49], leading to the inclusion of serotype 19A in the 13-valent vaccine. A high incidence of IPD due to serotype 19A has been associated with a limited number of clonal complexes (CC199, CC320, and CC276). Contrary to that, a study from Russia carried out by Mayanskiy et al. [50] demonstrated that local serotype distribution among non-invasive 19A-positive isolates can occur without vaccine pressure.

In our study, two isolates (1.8%) belonged to serotype 15A, which is not included in PCV-13. This serotype was previously associated with MDR and was isolated from most IPD cases in the post-PCV era in several post-industrial countries [51–53]. Our two serotype 15A isolates were PEN and macrolide susceptible and were ST2186, which was not previously associated with globally disseminated clones.

A single carriage isolate from our collection displayed serotype 35B, which is a non-vaccine serotype associated with high capacity for biofilm production [7]. The isolate was susceptible to PEN and macrolides, but showed resistance to TET and was associated with ST3185/none-CC in contrast to previous reports [54, 55]. The expansion of serotype 35B associated with both IPD and non-IPD cases in paediatric populations has been reported in several countries after the introduction of PCV-13 [52, 54].

### Resistance to antimicrobial agents

Since the pneumococcal disease is preceded by asymptomatic colonization, the distribution of antimicrobial resistance patterns in nasopharyngeal *S. pneumoniae* carriage strains may predict rates of resistance in invasive isolates [56, 57]. Rates of non-susceptibility among invasive and carriage isolates changed dramatically after the introduction of PCVs in industrialized countries [51–53, 58]. Furthermore, non-susceptibility to PEN in invasive pneumococcal isolates after the introduction of PCVs was strongly associated with an increased mortality rate in infants and children, as well as in the elderly [2]. The study found significantly higher rates of PEN and macrolide non-susceptibility than previously reported from Russia before the vaccine implementation [31, 34]. High rates of MDR carriage was discovered during the survey. Similar to the intermediate rate of carriage, an intermediate rate of PNSP was found. Treatment with antimicrobials 3 months before sampling was not a significant risk factor for carriage of PNSP in this cohort. A high concordance between non-susceptibility to PEN and macrolides and genotypes was also noted. Remarkably, a recently published study from Russia demonstrated a significant rise in resistance to oxacillin, erythromycin and clindamycin in disease-associated nasopharyngeal isolates in response to PCV-13 implementation. The growing resistance was explained by the expansion of MDR endemic clone ST143 with serotype 14 [59].

Contrary to the reported low rates of SXT consumption in the area [60] the study found a much higher rate of resistance to SXT than previously reported [31, 33, 34] and a high rate of non-susceptibility to TET. The 2006 all-Russia survey cited non-susceptibility rates of 43 and 53% to TET for the European and Asian parts of the country, respectively [34].

Rates of resistance are strongly associated with rates of antimicrobial consumption in local settings. According to the report from the European Surveillance of Antimicrobial Consumption (ESAC) network [61], the rates of outpatient antimicrobial consumption in Russia in 2006 were the lowest among all 33 participating countries. However, the low rates of antibiotic consumption at outpatient level do not agree with the present study. On the contrary it was found that children were intensively treated with antimicrobials prior to sampling. The low level of consumption published by ESAC should be regarded with caution due to possible bias in the reported sales and self-medication data [62].

### Results of MLST

We observed a high prevalence of various locally disseminated STs in the area. Although the Arkhangelsk region has rather low rates of migration and tourism and does not border any other countries, we found a close clonal relationship with the major globally disseminated pandemic clones, thus indicating possible import. Our study found ST1500 with serotype 23F to be associated with PEN non-susceptibility. A study carried out in Siberia found a high rate of ST1500 carriage isolates which were susceptible to PEN thus contrasting to the Arkhangelsk isolates [32]. Differences in the susceptibility profiles among strains within the country may suggest the acquisition of resistance in response to local antimicrobial prescribing practices. Besides, 1.2% of macrolide-resistant isolates were associated with ST2991 and ST3201. To the authors’ knowledge, these STs have never been associated with macrolide resistance before, which is also may be linked to a local treatment choice.

### Effects of PCV-13 vaccination

We found that PCV-13 could be effective against 67% of the pneumococcal population and thus reduce the majority of penicillin, macrolide, and multidrug-resistant strains as previously shown in other countries [63–66]. However, 1.2% of macrolide-resistant isolates were associated with non-vaccine serotypes 35F and 23A (35F-ST2991 and 23A-ST3201-CC346), that may replace the PCV-13-vaccine associated serotypes. ST2991 is a singleton not previously related to any clonal complexes, whereas ST3201 is part of the ST346-cluster, which has previously been linked to PEN resistance and several IPD-associated serotypes [67].

A high proportion of both penicillin and macrolide non-susceptible isolates in our study was found to be related to NT isolates. The recently published meta-analysis of estimated invasive disease potential for individual pneumococcal serotypes showed a low disease potential for non-serotypeable pneumococci [44]. However, all non-serotypeable isolates in our study were closely related and belonged to the ST344-cluster, the globally disseminated PMEN clone Norway^NT^-42, vaccination against which is so far unavailable.

The high effectiveness of PCVs against IPD in infants and young children has been proven in countries with well-established national surveillance. According to several reports [63–66] a sharp decrease of both carriage and IPD cases with vaccine-associated serotypes has occurred shortly after the introduction of PCVs with a subsequent decline in morbidity and mortality rates associated with these serotypes. It is too early to estimate the vaccine impact in Russia based on serotype distribution only. Reliable national and regional surveillance of invasive and non-invasive cases is needed to determine the effectiveness of the PCVs introduction and suggest strategies concerning vaccination schedules, the choice of PCV and vaccination coverage targets.

## Conclusions

The present study has documented several important aspects of the local pneumococcal epidemiology specific for the north of European Russia. The *S. pneumoniae* population was found to be highly diverse and had common features such as age-dependency, dominant serotypes and the presence of major epidemic clones. High rates of resistance were linked to high rates of antimicrobial consumption in the area. Clonal expansion of several globally distributed pandemic clones was identified in a remote part of north European Russia with low rates of migration and tourism. The effectiveness of PCV-13 introduction cannot easily be predicted. According to international experience, clonal expansion due to replacement by the globally disseminated disease-associated pandemic clones with non-PCV-13-serotypes can be expected. The authors propose setting up a high-quality population-based national/regional surveillance system for asymptomatic colonization and serotype-specific IPD rates for monitoring effects of the vaccination programme.

## Supplementary information

**Additional file 1: Table S1.** Odds ratio of *Streptococcus pneumoniae* nasopharygeal carriage by hypothesised risk factors. A study among children in kindergartens in Arkhangelsk Russia 2006, *N* = 438*.

## Data Availability

The data and materials are available on request from the corresponding author (Veronika Vorobieva Solholm Jensen, Dept. of Virus and Microbiological Special Diagnostics, Statens Serum Institute; Artillerivej 5, DK-2300 Copenhagen S, Denmark. Email:veronika.v.vorobieva@gmail.com), but restrictions apply under licence for the current study. The data may be publicity available upon reasonable request and with permission of the Northern State Medical University, Arkhangelsk, Russia.

## Abbreviations

BMI: Body Mass Index
CC: Clonal complex
CI: Confidence Interval
CIP: Ciprofloxacin
CLI: Clindamycin
CTX: Cefotaxime
CXM: Cefuroxime
cMLS: constitutive Macrolides, Lincosamides, Streptogramines resistance
DCCs: Daycare centers
DDD: Double-Disk Diffusions test
ERY: Erythromycin
ESAC: European Surveillance of Antimicrobial Consumption
EURIS: European Intervention Study
HIV: Human Immunodeficiency Virus
iMLS: inducible Macrolides, Lincosamides, Streptogramines resistance
IRRs: Incidence Rate Ratios
IPD: Invasive Pneumococcal Disease
LVX: Levofloxacin
MDR: Multidrug-resistance
MEM: Meropenem
MLST: Multilocus sequence typing
MNSP: Macrolide non-susceptible pneumococci
MXF: Moxifloxacin
NIP: National Immunization Programme
NOR: Norfloxacin
NT: Non-serotypeable
NWGA: Norwegian Working Group for Antibiotics
OR: Odds Ratio
OXA: Oxacillin
PCR: Polymerase Chain Reaction
PCV: Pneumococcal Conjugative Vaccine
PCV-7: 7-valent Pneumococcal Conjugate Vaccine
PCV-13: 13-valent Pneumococcal Conjugate Vaccine
PEN: Penicillin G
PMEN: Pneumococcal Molecular Epidemiology Network
PNSP: Penicillin non-susceptible pneumococci
SRGA: Swedish Reference Group for Antibiotics
ST: Sequence Type
SXT: Trimethoprim-sulfamethoxazole
TET: Tetracycline
WHO: World Health Organization
