# Supplementary material for: Epidemiological and molecular characterization of Streptococcus pneumoniae carriage strains in pre-school children in Arkhangelsk, northern European Russia, prior to the introduction of conjugate pneumococcal vaccines
Source: BMC Infect Dis. 2020 Apr 15;20:279. doi: 10.1186/s12879-020-04998-5 (PMC7161136; doi:10.1186/s12879-020-04998-5)
Supplement: Supplementary file 1 — Additional file 1: Table S1. Odds ratio of Streptococcus pneumoniae nasopharygeal carriage by hypothesised risk factors. A study among children in kindergartens in Arkhangelsk Russia 2006, N = 438*. [file 12879_2020_4998_MOESM1_ESM.docx]

| **Table S1. Odds ratio of *Streptococcus pneumoniae* nasopharygeal carriage by hypothesised risk factors. A study among children in kindergartens in Arkhangelsk Russia 2006, N = 438*** | | | | |
| --- | --- | --- | --- | --- |
|  | Univariable model | | Multivariable model | |
|  | OR | 95% CI | OR | 95% CI |
| *Sex*^1^ |  |  |  |  |
| Female | 1.00 |  | 1.00 |  |
| Male | 0.74 | 0.49-1.10 | 0.72 | 0.49–1.07 |
| *Age*, months^2^; |  |  |  |  |
| ≤18 | 1.00 |  | 1.00 |  |
| 19 - ≤36 | 0.089 | 0.005-0.483 | 0.084 | 0.005-0.456 |
| 37 - ≤59 | 0.143 | 0.008-0.760 | 0.134 | 0.007-0.716 |
| 60 - <84 | 0.210 | 0.011-1.135 | 0.195 | 0.011-1.062 |
| *Breastfeeding*^2^; |  |  |  |  |
| None | 1.00 |  | 1.00 |  |
| < 3 months | 1.18 | 0.479-3.279 | 1.26 | 0.480-3.173 |
| ≥ 3 months | 0.71 | 0.386-1.287 | 0.98 | 0.428-2.172 |
| *Number of rooms at home, apart from kitchen and bathroom*^1^ |  |  |  |  |
| 1-2 | 1.00 |  | 1.00 |  |
| ≥ 3 | 0.72 | 0.47-1.09 | 0.71 | 0.47-1.06 |
| *Average number of respiratory tract infections per year since birth*^1^ |  |  |  |  |
| Never or rare | 1.00 |  | 1.00 |  |
| Less than 6 times per year | 0.76 | 0.42-1.36 | 0.78 | 0.43-1.41 |
| More than 6 times per year | 0.59 | 0.24-1.41 | 0.56 | 0.23-1.38 |
| *Any acute illness within the last month*^1^ |  |  |  |  |
| Yes | 1.00 |  | 1.00 |  |
| No | 0.93 | 0.60-1.45 | 0.93 | 0.60-1.43 |
| *Any antibiotic treatment within the last 3 months*^1^ |  |  |  |  |
| Yes | 1.00 |  | 1.00 |  |
| No | 0.82 | 0.52-1.28 | 0.73 | 0.47-1.13 |
| *Number may vary due to missing. | | | | |

1. Multivariabel model adjusted for sex.
2. Multivariabel model adjusted for age.
